# Progressive Motor and Cognitive Dysfunction in Fahr's Disease: A Clinical Case Report

**DOI:** 10.7759/cureus.77969

**Published:** 2025-01-25

**Authors:** Mrooj M Almutairi, Zahraa J Ahmed, Masooma Y Ahmed, Shalan M Alshalan, Anas E Ahmed

**Affiliations:** 1 College of Medicine, King Abdulaziz University, Jeddah, SAU; 2 College of Medicine, Southeast University, Nanjing, CHN; 3 College of Medicine, Wenzhou Medical University, Wenzhou, CHN; 4 Emergency Medicine, King Khalid Hospital, Tabuk, SAU; 5 College of Medicine, Jazan University, Jazan, SAU

**Keywords:** antidepressants, basal ganglia calcification, cognitive decline, fahr disease, levodopa, movement disorder, neurodegenerative disorder, neuroimaging, psychiatric disturbances, rare diseases

## Abstract

Fahr's disease, or idiopathic basal ganglia calcification, is a rare neurodegenerative disorder characterized by the deposition of calcium salts in the basal ganglia, leading to a spectrum of neurological, cognitive, and psychiatric symptoms. This case report presents a 55-year-old female with a one-year history of progressive motor dysfunction, including tremors, dysarthria, and ataxia, along with cognitive decline and personality changes. Imaging studies revealed bilateral basal ganglia calcifications on both CT and MRI, confirming the diagnosis of Fahr's disease. The patient was started on symptomatic treatment with levodopa-carbidopa for motor symptoms and antidepressants for psychiatric disturbances. Despite some improvement in motor function, her cognitive decline continued to progress. The case emphasizes the importance of neuroimaging in diagnosing Fahr's disease and highlights the challenges of managing this rare disorder, particularly in its sporadic form. Treatment remains symptomatic, and a multidisciplinary approach is essential to address the diverse needs of patients. This case contributes to the understanding of Fahr's disease and underscores the need for further research into its pathophysiology and management.

## Introduction

Fahr's disease, also known as idiopathic basal ganglia calcification, is a rare neurodegenerative disorder characterized by the abnormal deposition of calcium salts within the basal ganglia, typically involving the globus pallidus, putamen, and caudate nucleus [1-3]. This condition can lead to a wide range of neurological symptoms, including movement disorders, cognitive decline, psychiatric disturbances, and, in some cases, seizures [2,3]. The exact cause of Fahr's disease remains unclear, although both genetic and environmental factors are believed to contribute. It is most commonly inherited in an autosomal dominant manner, with mutations in the SLC20A2, PDGFRB, and XPR1 genes implicated in familial cases. However, sporadic cases, where no family history is present, are also reported [2-5].

The disease typically manifests in adulthood, often between the ages of 30 and 60 years, with symptoms gradually worsening over time. Initial signs may include motor dysfunction such as tremors, rigidity, and bradykinesia, followed by cognitive decline and psychiatric symptoms [1,3]. Diagnosis is primarily based on imaging studies, with CT and MRI revealing characteristic bilateral basal ganglia calcifications. While there is no cure for Fahr's disease, symptomatic management is aimed at alleviating motor and psychiatric symptoms and improving the patient's quality of life. The prognosis is generally poor, with a progressive course leading to significant disability [3,5].

## Case presentation

A 55-year-old female with a past medical history significant for hypertension and type 2 diabetes mellitus presented to the neurology clinic with progressive motor and cognitive decline over the past year. The patient described experiencing difficulty with balance, tremors, and a noticeable decline in her ability to perform daily activities, including dressing and grooming. She also reported episodes of dysphagia and slurred speech. The patient’s family noted that her personality had changed, with increased irritability and apathy. No significant family history of neurological diseases was reported, and the patient denied any history of trauma, drug use, or alcohol consumption.

Upon physical examination, the patient appeared alert but mildly disoriented. Her speech was slow and dysarthric, and she exhibited mild tremors in both hands at rest. A cerebellar examination revealed moderate dysmetria with a positive finger-to-nose test bilaterally. Her gait was wide-based with mild ataxia. Muscle strength was normal in all extremities, but there was mild rigidity noted in both the upper and lower limbs, most pronounced in the arms. Reflexes were symmetrically increased in both the upper and lower extremities, with a positive Babinski sign bilaterally. Sensory examination was normal, and there was no evidence of any cranial nerve deficits.

**Figure 1:**
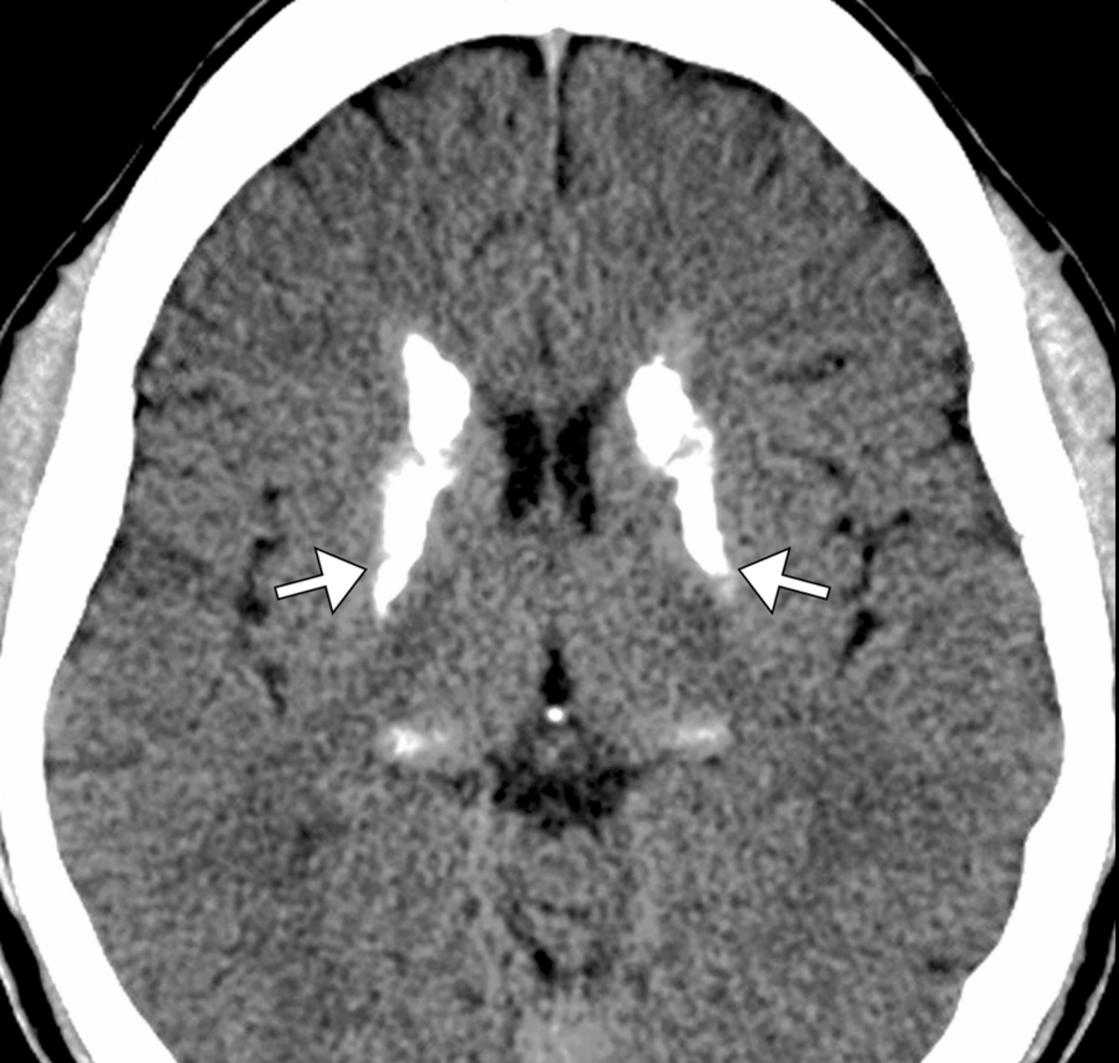
Axial CT of the brain showing bilateral dense calcification (arrows) of the basal ganglia.

**Figure 2:**
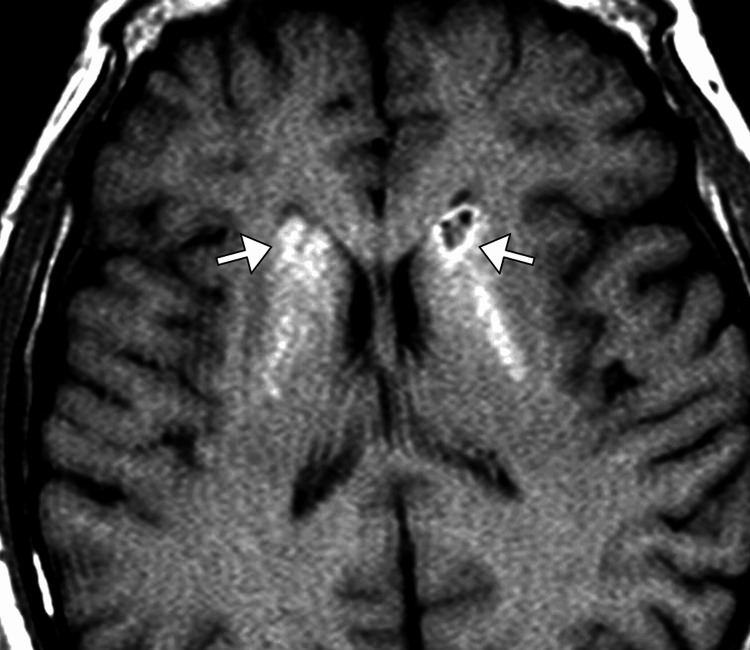
Axial T1-weighted MRI image of the brain showing bilateral dense calcification (arrows) of the basal ganglia.

A comprehensive work-up was initiated, beginning with laboratory investigations. Basic metabolic panel, liver function tests, and complete blood count were unremarkable. Thyroid function tests, vitamin B12 levels, and syphilis serology were also normal. Antinuclear antibody (ANA) and rheumatoid factor were negative, ruling out autoimmune causes. A CT scan of the brain was performed, revealing symmetrical calcifications in the basal ganglia, particularly in the globus pallidus and putamen. These findings were highly suggestive of Fahr's disease, a rare neurodegenerative disorder characterized by bilateral basal ganglia calcification (Figure 1). To confirm the diagnosis and rule out other possible causes, an MRI of the brain was subsequently obtained. This confirmed the CT findings, showing bilateral, extensive calcifications within the basal ganglia, as well as mild atrophy of the cerebral cortex (Figure 2).

The differential diagnosis for this patient included neurodegenerative diseases such as Parkinson’s disease, Huntington’s disease, and multiple system atrophy, given her motor symptoms and cognitive decline. However, the distinctive radiologic findings of basal ganglia calcifications on CT and MRI, along with her clinical presentation, were highly suggestive of Fahr's disease. Given the absence of a clear family history of similar neurological symptoms and the patient’s age, the diagnosis was confirmed as sporadic Fahr's disease.

Management was multidisciplinary, involving neurology, physical therapy, and speech therapy. Pharmacologically, the patient was started on levodopa-carbidopa to address the rigidity and bradykinesia, with moderate improvement in motor symptoms. Antidepressant therapy was also initiated to manage her irritability and apathy, which were affecting her quality of life. Speech therapy was provided to address her dysarthria and swallowing difficulties. Due to the progressive nature of the disease, there was no cure, and management was aimed at symptom control and improving functional capacity.

The patient was monitored closely during her hospital stay. During her admission, her motor symptoms stabilized, but cognitive decline continued to progress, as expected with Fahr's disease. The patient was discharged with a comprehensive care plan, including outpatient follow-up with neurology, physical therapy, and speech therapy. Genetic counseling was offered, but the patient declined testing for familial mutations associated with Fahr's disease. Regular follow-up appointments were scheduled to monitor disease progression and adjust management as necessary.

At her three-month follow-up, the patient’s motor symptoms remained stable with levodopa-carbidopa therapy, though cognitive impairment and speech difficulties had worsened. The family was provided with additional support resources to help with caregiving. Despite symptomatic management, the patient’s prognosis remained poor due to the progressive nature of Fahr's disease, and palliative care discussions were initiated. The patient and her family were educated on potential complications, including aspiration pneumonia and the risk of falls due to her ataxia, and were advised to implement safety measures at home.

## Discussion

Fahr's disease is a rare neurodegenerative disorder that presents with a unique constellation of neurological symptoms, primarily due to the deposition of calcium salts in the basal ganglia [2,4]. This case highlights the clinical complexity of Fahr's disease, where the patient exhibited a gradual onset of motor dysfunction, cognitive decline, and psychiatric changes, which are characteristic of the disease. The patient’s initial presentation of tremors, ataxia, and dysarthria, coupled with the progressive nature of her symptoms, strongly suggested a movement disorder, prompting further investigation. The characteristic bilateral basal ganglia calcifications seen on neuroimaging were key to establishing the diagnosis, in accordance with the clinical guidelines for diagnosing Fahr's disease [4-8].

One of the notable aspects of this case was the lack of a family history of neurological disease. Although familial forms of Fahr's disease have been well documented, the sporadic nature of this patient’s presentation emphasizes the fact that Fahr's disease can also occur in individuals without a genetic predisposition [1-4]. Genetic mutations, including those in the SLC20A2, PDGFRB, and XPR1 genes, have been implicated in familial cases, but the absence of such mutations in sporadic cases suggests that other environmental or genetic factors may contribute to the disease process. This highlights the need for further research into the pathogenesis of Fahr's disease, particularly in sporadic cases where no clear genetic link can be identified [2-6].

The patient's cognitive decline, which worsened over time, is consistent with the progressive nature of Fahr's disease. Cognitive impairment is commonly observed in these patients, with many experiencing dementia-like symptoms. Studies have shown that the severity of cognitive decline often correlates with the extent of basal ganglia calcifications [2,5]. While cognitive and psychiatric symptoms such as irritability, depression, and personality changes are frequently reported, the progression of these symptoms can vary widely among individuals. In this case, the patient's personality changes and apathy were significant and impactful on her quality of life, further complicating the management of the disease. It is essential to recognize and address the psychiatric manifestations of Fahr's disease, as they can significantly affect both the patient and their family, requiring a multidisciplinary approach to care [1,4].

The management of Fahr's disease remains largely symptomatic, as no specific treatment has been identified to halt or reverse the progression of the disease. Levodopa, a common treatment for movement disorders such as Parkinson's disease, was used in this case to alleviate the patient's rigidity and bradykinesia [2-4]. While the patient showed moderate improvement in motor symptoms, this improvement was not sustained, which aligns with the understanding that Fahr's disease does not always respond predictably to dopaminergic treatment. This is in contrast to Parkinson’s disease, where levodopa is often highly effective. The patient’s response to levodopa also highlights the complexity of managing Fahr's disease, as there is no consensus on the optimal pharmacologic treatment. Other medications, including antidepressants, may be used to manage psychiatric symptoms, as was the case for this patient. However, the overall prognosis remains poor, and treatment remains primarily supportive [1-5].

## Conclusions

In conclusion, Fahr's disease is a rare and progressive neurodegenerative disorder that presents with a diverse array of symptoms, including movement abnormalities, cognitive decline, and psychiatric disturbances, which can significantly impact a patient’s quality of life. While familial cases are well-established, sporadic cases, such as the one presented here, emphasize the complexity and variability in disease presentation and pathogenesis. Neuroimaging plays a crucial role in diagnosis, with basal ganglia calcifications serving as a hallmark feature. Although treatment remains symptomatic, focusing on alleviating motor and psychiatric symptoms, the prognosis remains poor due to the progressive nature of the disease. A multidisciplinary approach, including neurological, physical, and psychiatric care, is essential for optimizing patient outcomes and providing support to both the patient and their family. This case highlights the importance of early recognition and a tailored management plan, although challenges in disease progression and treatment response remain.
